# Development of a Prognostic Predictive Model for Stage III Non-Small Cell Lung Cancer using PET-CT Radiomics Based on Multi-Center and Heterogeneous Data and External Validation

**DOI:** 10.12669/pjms.42.5.14183

**Published:** 2026-05

**Authors:** Jianyang Zhang, Yujing Hu, Congna Tian, Xinchao Zhang, Yanzhu Bian

**Affiliations:** 1 Jianyang Zhang, Hebei Medical University, Shijiazhuang 050000, Hebei, China. Department of Nuclear Medicine, Hebei General Hospital, Shijiazhuang 050000, Hebei, China; 2 Yujing Hu, Hebei Medical University, Shijiazhuang 050000, Hebei, China. Department of Nuclear Medicine, Hebei General Hospital, Shijiazhuang 050000, Hebei, China; 3 Congna Tian, Hebei Medical University, Shijiazhuang 050000, Hebei, China. Department of Nuclear Medicine, Hebei General Hospital, Shijiazhuang 050000, Hebei, China; 4 Xinchao Zhang, Hebei Medical University, Shijiazhuang 050000, Hebei, China. Department of Nuclear Medicine, Hebei General Hospital, Shijiazhuang 050000, Hebei, China; 5 Yanzhu Bian, Hebei Medical University, Shijiazhuang 050000, Hebei, China. Department of Nuclear Medicine, Hebei General Hospital, Shijiazhuang 050000, Hebei, China

**Keywords:** ^18^F-FDG, PET/CT, Radiomics, Stage III Non–small cell lung cancer, Multicenter study, Overall survival,

## Abstract

**Objective:**

To compare the prognostic performance of models integrating PET/CT radiomics with metabolic parameters for predicting overall survival in patients with stage III NSCLC using multi-center retrospective data.

**Methodology:**

In this retrospective, comparative study, we analyzed data from 165 stage III NSCLC patients (from 37 centers in The Cancer Imaging Archive) and an independent external cohort of 48 patients from Hebei General Hospital (2022-2025). Radiomic features and metabolic parameters were extracted from baseline PET/CT images. Feature selection and cross-validation were performed to construct and compare multiple prediction models. Model performance was evaluated in training, validation, and external test cohorts using discrimination (AUC), calibration (Brier score), clinical net benefit (Decision Curve Analysis), and Kaplan-Meier survival analysis. Shapley additive explanations (SHAP) were applied to interpret the final model.

**Results:**

Five prediction models were developed and compared. The model combining PET radiomic features and whole-body metabolic tumor volume (MTVwb), termed the Combined_A model, demonstrated the best and most stable performance, with AUCs of 0.782, 0.775, and 0.778 in the training, validation, and external test cohorts, respectively. In the external test cohort. Combined_A also showed the lowest Brier score (Brier score: 0.188) and greater net clinical benefit. Kaplan-Meier analysis confirmed significant survival stratification (log-rank P= 0.002). SHAP analysis identified wavelet-HHL_GLSZM_Small Area Low Gray Level Emphasis and log first order Skewness as key prognostic features.

**Conclusion:**

In this retrospective comparative study, a model integrating PET radiomics with MTVwb provided robust and generalizable survival prediction for stage III NSCLC. These findings support its potential for personalized risk stratification, warranting further prospective validation.

**Registration No.:** (ClinicalTrials.gov: NCT00083083).

## INTRODUCTION

Lung cancer remains the leading cause of cancer-related mortality,^1^ Stage III non-small-cell lung cancer (NSCLC) is clinically heterogeneous, and existing prognostic systems have limited ability to accurately stratify patient risk.^2^ ^18F-FDG PET/CT is an essential imaging modality for staging and restaging NSCLC, particularly in evaluating mediastinal lymph node involvement.^3^ Current TNM staging and conventional PET metabolic parameters show limited ability to capture tumor heterogeneity at the individual level.^4^,^5^ PET/CT-based radiomics has emerged as a promising approach for prognostic modeling; however, most existing studies are single-center and lack external validation.^6^ Therefore, this multi-center study aimed to develop and externally validate PET/CT radiomics-based prognostic models for overall survival in stage III NSCLC patients.

## METHODOLOGY

This comparative retrospective study included patients from two cohorts. The primary cohort was derived from the ACRIN 6668/RTOG 0235 multicenter clinical trial^7^ (ClinicalTrials.gov: NCT00083083), which enrolled patients with locally advanced NSCLC who underwent baseline FDG PET or PET/CT imaging prior to concurrent chemoradiotherapy. Imaging data were obtained from The Cancer Imaging Archive.^8^ Among 251 enrolled patients, cases with poor image quality, PET-only scans, stage IIB disease, or incomplete data were excluded. Finally, 165 stage III NSCLC patients from 37 centers were included and randomly divided into training and validation cohorts at a 6:4 ratio. An independent external test cohort consisted of 48 patients with stage III NSCLC who were retrospectively identified at Hebei General Hospital between January 2022 and December 2025.

### Ethical Approval:

The study was approved by the Institutional Ethics Committee of Hebei General Hospital (No.:[2023]075; date: October 19, 2023).

### Inclusion criteria:

- Histologically confirmed NSCLC;
- Baseline FDG PET/CT imaging performed prior to any treatment;
- Stage III disease according to the 8th edition of the AJCC TNM staging system;
- Complete clinical and follow-up data;
- The external cohort, a minimum follow-up of 12 months.

### Exclusion criteria:

- Poor image quality or incomplete imaging data;
- Prior history of other malignancies;
- Receipt of any anticancer treatment before PET/CT;
- Missing essential clinical variables;
- Concurrent severe medical conditions that could compromise survival assessment (e.g., uncontrolled infection, severe organ dysfunction).

### PET/CT Imaging and Tumor Segmentation:

In the ACRIN cohort, PET/CT scans were acquired using qualified scanners according to standardized protocols.^9^ Patients received an intravenous injection of ^18F-FDG, and whole-body imaging was performed approximately 50–70 minutes after injection. The external cohort underwent PET/CT imaging using standard clinical acquisition and reconstruction protocols.

Pretreatment PET and CT images were registered, and primary tumor lesions were semi-automatically segmented on PET images and manually refined on CT images. Two independent observers performed tumor delineation. Radiomic features with an intraclass correlation coefficient (ICC) below 0.75 were excluded to ensure reproducibility.

### Radiomic Feature Extraction and Selection:

To reduce inter-center variability, images were resampled to a uniform voxel size of 2 × 2 × 2 mm³ and discretized using fixed bin widths. Radiomic features were extracted using PyRadiomics in accordance with Image Biomarker Standardization Initiative guidelines,^10^ including first-order statistics, shape features, texture features, and filtered features. Missing values were imputed using median values. Z-score normalization was applied to all features. Highly correlated features (Pearson correlation coefficient > 0.7) were removed to reduce redundancy. Gradient boosting decision trees were used to select informative radiomic features.^11^

Clinical and metabolic variables were analyzed using univariable and multivariable Cox proportional hazards regression to identify variables associated with OS. These analyses were used only for variable screening and not for model construction.

### Model Development and Evaluation:

Overall survival (OS) was defined as the time from baseline PET/CT imaging to death from any cause or last follow-up. Patients alive at the last follow-up were censored. Given the limited number of long-term survivors, OS was dichotomized based on five-year survival status for model development. To address class imbalance, the synthetic minority over-sampling technique was applied to the training cohort.^12^ Logistic regression models were constructed using selected radiomic features with or without clinical variables. Five-fold cross-validation was performed in the training set.

Model performance was evaluated using receiver operating characteristic curves and area under the curve (AUC) values. Calibration was assessed using calibration curves, Brier scores, and expected calibration error. Decision curve analysis was conducted to evaluate clinical utility. The optimal probability threshold was determined using Youden’s index.

### Model Interpretation and Statistical Analysis:

Kaplan-Meier survival curves and log-rank tests were used to assess survival differences between model-defined risk groups. Shapley additive explanations were applied to evaluate feature contributions to model predictions. All statistical analyses were performed using R and SPSS software. All tests were two-sided, and P < 0.05 was considered statistically significant.

## RESULTS

The demographic and clinical characteristics of patients in the ACRIN and Hebei General Hospital (HBGH) cohorts are summarized in Table-I. All patients had stage III NSCLC. Treatment regimens differed between cohorts, with uniform concurrent chemoradiotherapy in the ACRIN cohort and heterogeneous real-world treatments in the HBGH cohort. A significant difference in progression-free survival (PFS) was observed between cohorts (8.0 vs. 18.5 months, P < 0.001); therefore, subsequent analyses focused on overall survival (OS). Apart from body weight, no significant differences were observed in baseline clinical variables.

**Table-I:** Summary of Demographic and Clinical Data from Three Study Cohorts.

Demographics/Clinical characteristics	ACRIN 6668		HBGH	p Values
	Train(n=99)	Test(n=66)	External test(n=48)	
Age, years	64.14±10.027	65.58±9.152	64.77±10.111	0.654
Sex, male	65(65.7%)	44(66.7%)	38(79.2%)	0.131
Height, cm	170.44±10.23	169.80±12.58	168.13±7.05	0.450
Weight, kg	76.70±17.49	76.52±13.79	66.21±12.07*	0.000*
Diameters, cm	41(14-116)	41(13-100)	39(7-111)	0.835
SUVmax	15.43(1.61--53.22)	13.05(0.60-41.31)	13.97(1.70-39.30)	0.147
SUVpeak	14.57(1.21-53.22)	12.41(0.50-41.31)	16.59(1.10-33.77)	0.135
MTVtumor	16.73(0.37-498.95)	21.93(0.64-487.40)	12.59(1.87-571.53)	0.735
TLGtumor	179.20(1.46-6160.52)	173.16(0.30-3866.92)	169.74(4.40-2826.16)	0.355
MTVwb	43.35(1.69-504.82)	40.67(3.05-783.76)	38.73(6.65-603.52)	0.108
TLGwb	381.60(17.46-6258.52)	356.57(13.30-3962.92)	237.89(12.40-3842.83)	0.514
Patient’s Vital Status, Dead	71(71.7%)	48(72.7%)	31(64.6%)	0.113
OS, month	23(2-60)	16(1-60)	23(1-89)	0.383
PFS, month	9(2-60)	8(1-60)	18.5(1-73)*	0.005*

* p < 0.05 indicates statistical significance. Abbreviations: SUVmax: Maximum Standardized Uptake Value; MTVtumor: Metabolic Tumor Volume (tumor); TLGtumor: Total Lesion Glycolysis (tumor); SUVpeak: Peak Standardized Uptake Value; MTVwb: Metabolic Tumor Volume (whole body); TLGwb: Total Lesion Glycolysis (whole body); OS: Overall Survival; PFS: Progression-Free Survival.

A total of 1,130 radiomic features were extracted from pretreatment PET and CT images. After excluding features with low reproducibility (ICC < 0.75) and redundant features, six PET and three CT radiomic features were selected using gradient boosting decision trees (Supplementary Table-I). Univariable and multivariable Cox regression analyses identified whole-body metabolic tumor volume (MTVwb) as the only independent clinical factor associated with OS (HR = 1.012, 95% CI: 1.002–1.021, P = 0.043). ROC analysis using MTVwb alone yielded limited predictive performance across the training, validation, and external test cohorts (AUCs: 0.683, 0.720, and 0.588, respectively).

**Supplemental Table-I:** Radiomic Features Selected from PET and CT Images and Corresponding Model Coefficients.

Modality	Feature Name	PET Coefficient	CT Coefficient	PET-CT Coefficient
PET	original_shape_Sphericity	-0.4268	–	-0.3243
PET	original_glszm_SmallAreaLowGrayLevelEmphasis	0.4703	–	0.4371
PET	log-sigma-2-0-mm-3D_firstorder_Skewness	0.7505	–	0.6938
PET	wavelet-HHL_glszm_SmallAreaLowGrayLevelEmphasis	-1.0572	–	-0.9991
PET	wavelet-HHH_firstorder_Uniformity	-0.4514	–	-0.3319
PET	wavelet-HHH_glszm_ZoneVariance	2.5700	–	2.4045
CT	wavelet-HLL_glcm_ClusterShade	–	0.4048	0.2513
CT	wavelet-HHL_firstorder_Skewness	–	-0.1261	-0.1311
CT	wavelet-HHH_glszm_SizeZoneNonUniformityNormalized	–	0.6165	0.1970

*Note:* Coefficients were derived from regularized logistic regression.

Blank cells (denoted by “–”) indicate the feature was not selected in the corresponding model.

**Fig.1:**
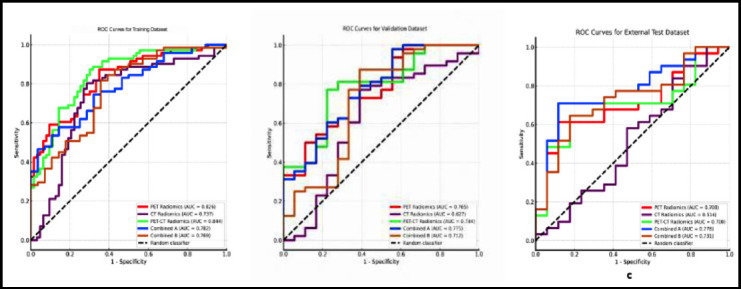
ROC curves for the five models in the training. (A), validation(B), and external test(C) datasets.

Five logistic regression-based models were constructed: PET_radiomics, CT_radiomics, PET/CT_radiomics, Combined_A, and Combined_B. In the training and validation cohorts, PET/CT_radiomics and Combined_A demonstrated comparable performance. In the external test cohort, Combined_A achieved the highest AUC (0.778), followed by Combined_B (0.731) and PET_radiomics (0.708), whereas CT_radiomics showed poor discrimination (AUC = 0.514) (Fig.1 and Suplementary Table-II). DeLong’s test confirmed significant differences between CT_radiomics and the other models.

**Fig.2:**
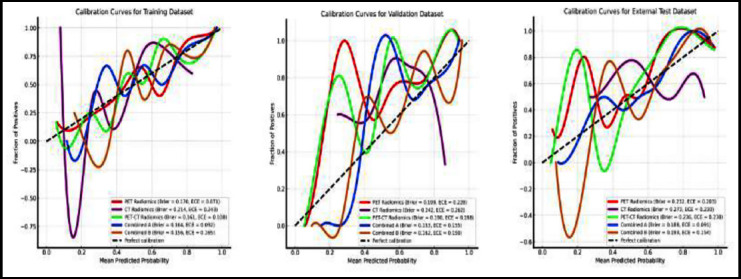
Calibration curve for the five models in the training. (A), validation(B), and external test(C) datasets.

**Fig.3:**
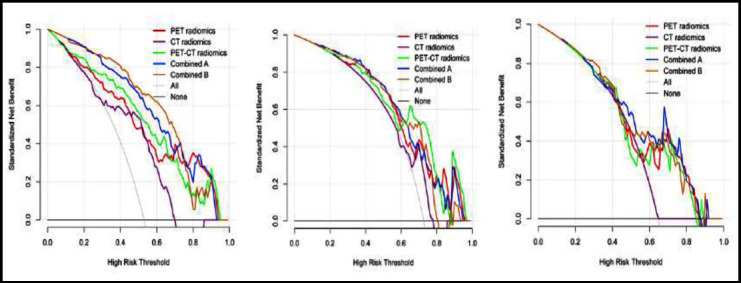
Decision Curve Analysis (DCA) for the five models in the training. (A), validation(B), and external test(C) datasets.

In the external test cohort, Combined_A demonstrated the lowest Brier score (0.188) and expected calibration error (0.080), indicating superior calibration (Fig.2). Decision curve analysis showed greater net benefit across clinically relevant thresholds (Fig.3).

**Fig.4:**
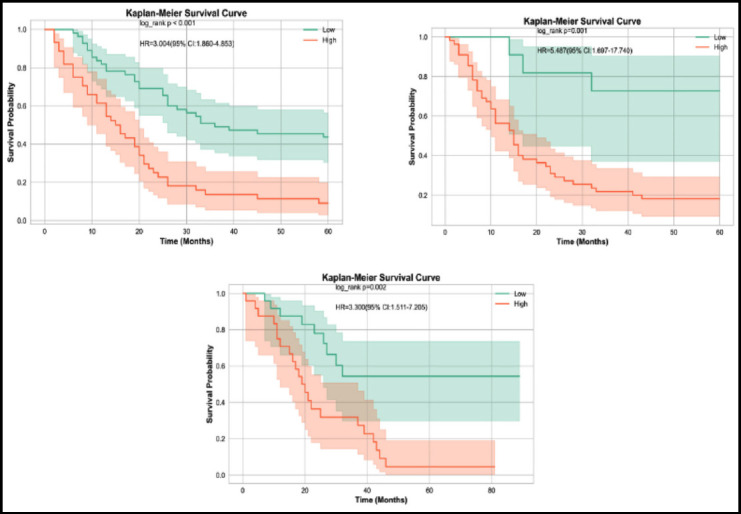
Kaplan-Meier survival analysis for the Combined. A model in the training (A), validation(B), and external test(C) datasets.

**Fig.5:**
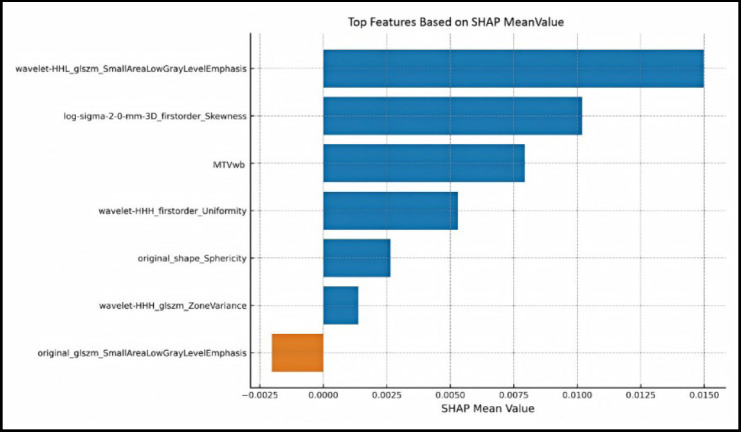
Features based on SHAP mean values for the Combined_A model.

Kaplan-Meier analysis showed significant OS differences between high- and low-risk groups across all cohorts (log-rank P < 0.01)(Fig.4). SHAP analysis identified wavelet-HHL GLSZM Small Area Low Gray Level Emphasis and log-sigma-2-0-mm-3D_firstorder_Skewness as the most influential features (Fig.5 and Supplementary Table-III).

**Supplemental Table-II:** The Diagnostic Performance of the Five Models for in the Three Cohorts.

Cohorts	Model	AUC(95% CI)	Specificity	Precision	F1 score	Recall
Training cohort	PET_radiomics	0.826(0.752-0.887)	0.698	0.736	0.741	0.746
	CT_radiomics	0.737(0.645-0.824)	0.714	0.757	0.772	0.789
	PET-CT_radiomics	0.844(0.776-0.908)	0.73	0.77	0.786	0.803
	Combined_A	0.782(0.682-0.87)	0.321	0.776	0.846	0.93
	Combined_B	0.769(0.658-0.874)	0.357	0.786	0.852	0.93
Validation cohort	PET_radiomics	0.765(0.617-0.878)	0.667	0.846	0.759	0.688
	CT_radiomics	0.627(0.443-0.78)	0.611	0.821	0.736	0.667
	PET-CT_radiomics	0.784(0.649-0.899)	0.778	0.9	0.818	0.75
	Combined_A	0.775(0.629-0.894)	0.444	0.815	0.863	0.917
	Combined_B	0.712(0.537-0.855)	0.389	0.8	0.854	0.917
External testing cohort	PET_radiomics	0.708(0.545-0.851)	0.765	0.826	0.704	0.613
	CT_radiomics	0.514(0.319-0.689)	0.529	0.636	0.528	0.452
	PET-CT_radiomics	0.7(0.539-0.836)	0.706	0.8	0.714	0.645
	Combined_A	0.778(0.642-0.911)	0.412	0.73	0.794	0.871
	Combined_B	0.731(0.571-0.863)	0.412	0.706	0.738	0.774

**Supplementary Table-III:** SHAP Mean Values and Absolute SHAP Mean Values for Radiomic Features in Model Combined_A.

Feature Name	SHAP Mean Value	Absolute SHAP Mean Value
wavelet-HHL_glszm_SmallAreaLowGrayLevelEmphasis	0.014986	0.183174
log-sigma-2-0-mm-3D_firstorder_Skewness	0.010189	0.054198
wavelet-HHH_glszm_ZoneVariance	0.001381	0.050400
original_shape_Sphericity	0.002644	0.043633
MTVwb	0.007928	0.027594
original_glszm_SmallAreaLowGrayLevelEmphasis	-0.002020	0.019812
wavelet-HHH_firstorder_Uniformity	0.005297	0.013678

***Note:*** SHAP mean values represent the average contribution of each feature to the model’s predictions, while absolute SHAP mean values represent the magnitude of each feature’s effect regardless of sign.

## DISCUSSION

This study demonstrates that PET/CT-based radiomics provides prognostic value for overall survival prediction in patients with stage III NSCLC using multi-center data and external validation. Given the pronounced heterogeneity of stage III disease and the limited discriminative ability of TNM staging,^13^ improved risk stratification at the individual level remains clinically important. Our findings indicate that integrating PET radiomic features with whole-body metabolic tumor volume (MTVwb) enhances survival prediction and enables effective patient stratification.

The prognostic role of conventional PET metabolic parameters remains debated.^14^,^15^ Although volumetric parameters such as MTV and TLG are considered more robust indicators of tumor burden,^16^,^17^ MTVwb alone showed limited predictive performance in our study, particularly in the external cohort. These results suggest that global metabolic burden alone does not sufficiently capture tumor heterogeneity in stage III NSCLC.

Radiomics offers a quantitative means to characterize intratumoral heterogeneity.^18^ In this study, PET radiomics-based models consistently outperformed CT radiomics, especially in the external validation cohort. The limited performance of CT radiomics may be attributable to segmentation challenges in advanced disease, where lymph node involvement, atelectasis, and obstructive pneumonia can obscure tumor boundaries. These findings support the greater relevance of PET-derived features in reflecting tumor biology in stage III NSCLC.

Among the evaluated models, the Combined_A model demonstrated the most robust and stable performance across all cohorts, achieving superior discrimination, calibration, and clinical net benefit. Notably, its consistent performance in the external test cohort, which included patients receiving heterogeneous real-world treatments, highlights its potential clinical applicability beyond controlled trial settings.

SHAP analysis identified wavelet-HHL_GLSZM_Small Area Low Gray Level Emphasis and firstorder_Skewness as key contributors to survival prediction, reflecting intratumoral heterogeneity and asymmetric metabolic activity.^19^,^20^ While the biological correlates of these features require further investigation, their importance supports the prognostic relevance of radiomic heterogeneity in advanced NSCLC.

### Limitations.

First, the sample size is relatively modest, which may limit statistical power and generalizability. Second, the retrospective design introduces inherent selection bias, despite the use of multi-center data. Third, heterogeneity in imaging protocols and equipment across centers could introduce variability; although we attempted to mitigate this by image resampling and feature selection based on ICC, more advanced harmonization techniques such as ComBat were not applied.^21^ Fourth, the absence of molecular biomarkers (e.g., EGFR mutation, PD-L1 expression) precludes integration of genomic data, which might improve prognostic accuracy. Fifth, due to the retrospective nature, information on comorbidities such as asthma or pulmonary edema was not available; therefore, these conditions were not excluded, and their potential impact on survival cannot be fully assessed. Sixth, the dichotomization of overall survival at five years simplifies the time-to-event nature of the outcome; future studies should employ time-to-event models (e.g., Cox proportional hazards) directly. Finally, external validation was performed at a single institution, and further validation in larger, more diverse populations is warranted.

## CONCLUSIONS

This study demonstrates the strong prognostic value of PET/CT-based radiomics for predicting overall survival in stage III NSCLC. By integrating PET radiomic features with whole-body metabolic tumor volume (MTVwb), the model provides robust and generalizable survival predictions, as evidenced by its consistent performance across multi-center datasets. These findings underscore the model’s potential to support individualized risk stratification and guide personalized treatment decision-making for patients with stage III NSCLC.

### Authors’ Contributions:

**JZ:** Data curation, Investigation, Methodology, and Writing-original draft, prepared figures 1-5, and is responsible and accountable for the accuracy or integrity of the work.

**YB:** Conceptualization, Project administration, Supervision, critical review & editing.

**YH:** Funding acquisition, Methodology, and Supervision. Critical review

**CT:** Data curation, Methodology, Software, and Validation.

**XZ:** Data curation and Writing-review & editing. All authors reviewed the manuscript.

All authors have read and approved the final manuscript.
